# Supplementary material for: k-mer manifold approximation and projection for visualizing DNA sequences
Source: Genome Res. 2025 May;35(5):1234–46. doi: 10.1101/gr.279458.124 (PMC12047656; doi:10.1101/gr.279458.124)
Supplement: Supplement 8 [file Supplemental_Data_1.zip › Supplemental_Data_1/POU3F2_TGTTCA40NCTG_AI_5/POU3F2_TGTTCA40NCTG_AI_5_tSNE.pdf]

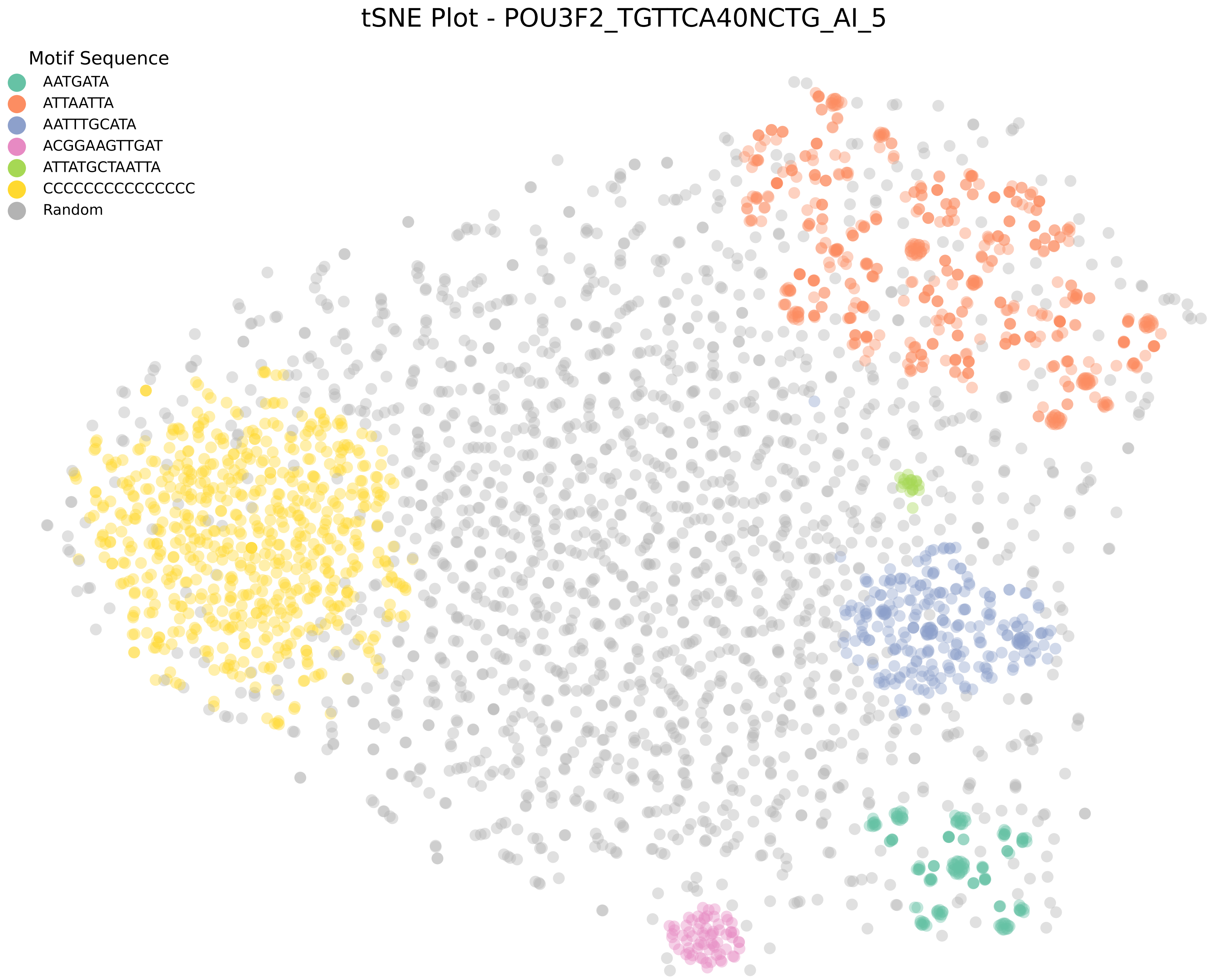

tSNE Plot - POU3F2_TGTTCA40NCTG_AI_5
Motif Sequence
AATGATA
ATTAATTA
AATTTGCATA
ACGGAAGTTGAT
ATTATGCTAATTA
CCCCCCCCCCCCCCC
Random
